# Plasma membrane order and fluidity are diversely triggered by elicitors of plant defence

**DOI:** 10.1093/jxb/erw284

**Published:** 2016-07-18

**Authors:** Roman Sandor, Christophe Der, Kevin Grosjean, Iulia Anca, Elodie Noirot, Nathalie Leborgne-Castel, Jan Lochman, Françoise Simon-Plas, Patricia Gerbeau-Pissot

**Affiliations:** ^1^Department of Biochemistry, Faculty of Science, Masaryk University, Kotlářská 2, 61137 Brno, Czech Republic; ^2^Agroécologie, AgroSup Dijon, CNRS, INRA, Université Bourgogne Franche-Comté, F-21000 Dijon, France

**Keywords:** Cryptogein mutants, elicitors, fluidity, membrane order, plant defence, plasma membrane, reactive oxygen species, signalling.

## Abstract

Although plants are exposed to a great number of pathogens, they usually defend themselves by triggering mechanisms able to limit disease development. Alongside signalling events common to most such incompatible interactions, modifications of plasma membrane (PM) physical properties could be new players in the cell transduction cascade. Different pairs of elicitors (cryptogein, oligogalacturonides, and flagellin) and plant cells (tobacco and Arabidopsis) were used to address the issue of possible modifications of plant PM biophysical properties induced by elicitors and their links to other events of the defence signalling cascade. We observed an increase of PM order whatever the elicitor/plant cell pair used, provided that a signalling cascade was induced. Such membrane modification is dependent on the NADPH oxidase-mediated reactive oxygen species production. Moreover, cryptogein, which is the sole elicitor able to trap sterols, is also the only one able to trigger an increase in PM fluidity. The use of cryptogein variants with altered sterol-binding properties confirms the strong correlation between sterol removal from the PM and PM fluidity enhancement. These results propose PM dynamics as a player in early signalling processes triggered by elicitors of plant defence.

## Highlight

The ROS-dependent increase of plasma membrane order is a generic event triggered by elicitors of plant defence, whereas fluidity enhancement is specifically triggered by cryptogein, a sterol-carrier elicitin

## Introduction

Induction of plant defence against pathogens can be mediated by elicitors, namely compounds able to trigger structural, biochemical, and/or molecular responses associated with the expression of plant disease resistance in host plants. Biotic elicitors belong to various biochemical classes corresponding to either evolutionarily conserved microbial signatures secreted by both non-pathogenic and pathogenic microorganisms, termed microbe/pathogen-associated molecular patterns (M/PAMPs), or plant endogenous molecules deriving from cell wall breakdown products or corresponding to released soluble peptides, referred to as damage-associated molecular patterns (DAMPs) (Bartels and Boller, 2015). Elicitors are perceived by plant recognition systems and activate signal transduction cascades (Boller and Felix, 2009), including initial events localized at the plant plasma membrane (PM) (for recent examples, see Matsui *et al*., 2014; Maruta *et al.*, 2015).

The host PM can thus serve as a critical barrier recognizing microbes, even if the perception system itself is unknown for numerous plant cell–elicitor pairs. Some cell surface-localized immune receptors such as pattern recognition receptors (PRRs), which detect the presence of a pathogen threat, are anchored in the PM (Zipfel, 2014). Among hundreds of sequences coding for putative PRRs, only a few PRR/M/PAMP pairs have been characterized. The paradigmatic pair is the LRR-RLK (leucine-rich repeat receptor-like kinase) FLS2 (Flagellin-Sensing2) from *Arabidopsis thaliana* and the flg22 peptide from the bacterial flagellin (Gomez-Gomez *et al*., 1999; Chinchilla *et al.*, 2006). In addition, to mediate recognition of elicitins that are oomycete PAMPs, both a PM-associated high affinity binding site and a cell surface-associated receptor-like protein ELR (elicitin response) were reported in tobacco (Wendehenne *et al.*, 1995; Bourque *et al.*, 1999) and potato (Du *et al.*, 2015), respectively. Cryptogein, an elicitin secreted by *Phytophthora cryptogea*, exhibits a structure consisting of one β-sheet, five α-helices, and one ω-loop, and acts as a sterol transfer protein, able to load up sterols from the host PM and to deliver them to the oomycete membrane (Mikes *et al.*, 1997). After binding of sterol into the cavity, a shift in the ω-loop conformation of cryptogein was observed (Boissy *et al.*, 1999). The formation of such a sterol–cryptogein complex is necessary for effective interactions of cryptogein with high-affinity binding sites on the tobacco PM (Osman *et al.*, 2001). A recent study assumed the possible involvement of a third partner complex in this binding process (Dokladal *et al.*, 2012). In the case of DAMPs such as oligogalacturonides (OGs), the plant perception system is still questioned, although members of the wall-associated kinase (WAK) family are candidate receptors (Brutus *et al.*, 2010).

After the recognition step, early responses induced by M/P/DAMPs largely overlap and a signalling cascade consisting of a complex network of second events, including protein phosphorylations, calcium influx, cytosol acidification, etc, in turn triggers defence reactions (Batz *et al.*, 1998; Lebrun-Garcia *et al.*, 1999; Macho and Zipfel, 2014; Bigeard *et al.*, 2015). Reactive oxygen species (ROS) production is a relevant example of conserved signalling output during plant immunity (Nanda *et al.*, 2010; Choudhury *et al.*, 2013). Among many roles, ROS have been proposed to act as antimicrobial agents, cross-linkers of the plant cell wall to block pathogen ingress, or local and systemic players to trigger immune responses, such as defence-related gene expression (Lamb and Dixon, 1997; Suzuki *et al.*, 2011; Nathan and Cunningham-Bussel, 2013). In plants, PM-localized NADPH oxidases are very important early-stage ROS-producing enzymes and belong to the respiratory burst oxidase homologue (RBOH) family. In *Arabidopsis thaliana*, AtRbohD and AtRbohF isoforms are responsible for the PAMP-induced ROS burst, but with a prevalent role for AtRbohD (Torres *et al.*, 2002; Pogany *et al.*, 2009). Likewise, silencing of the *NtRbohD* gene in tobacco plants induces an inhibition of cryptogein-induced ROS accumulation (Simon-Plas *et al.*, 2002). Moreover, *A. thaliana* mutants affected in NADPH oxidase-mediated ROS production displayed enhanced leaf necrosis after exposure to the biotrophic oomycete *Peronospora parasitica*, but diminished cell death symptoms after inoculation with an avirulent strain of *Pseudomonas syringae* (Torres *et al.*, 2002). *Nicotiana benthamiana* silenced plants were, in turn, compromised in the *Phytophthora infestans*-mediated activation of disease resistance (Yoshioka *et al.*, 2003). In *Nicotiana tabacum*, elicitation with cryptogein induced severe ultrastructural damage in mesophyll cells of a mutant affected in ROS production, whereas the morphology of the wild type was not affected, suggesting that the oxidative burst is mainly associated with the protection of the plant cell (Lherminier *et al.*, 2009). These diverse effects suggest the involvement of ROS at several steps of the complex signalling pathways of plant defence responses (Torres, 2010).

Although dynamic adjustment of PM physical properties emerges as the primary determinant of plant cell survival in fluctuating conditions (Vaultier *et al.*, 2006; Konigshofer *et al.*, 2008), its possible induction after sensing of the invading pathogen by plant cells has been poorly investigated. Two main parameters deserve to be used to characterize membrane biophysics: fluidity as a measure of the rotational and diffusional motions of molecules within the membrane; and order, comprising structure, microviscosity, and membrane phases (Rilfors *et al.*, 1984; van der Meer *et al.*, 1984; Bloom *et al.*, 1991). The order refers to physical segregation induced by lipid self-association into lipid bilayers, wherein a liquid-ordered (L_o_) phase co-exists with a liquid-disordered (L_d_) phase (Veatch and Keller, 2005; Gaus *et al.*, 2006; Klymchenko *et al.*, 2009; Heberle *et al.*, 2011). Previous work using cryptogein and flg22 suggested that both modifications of PM physical properties could be induced differently by elicitor treatment (Gerbeau-Pissot *et al.*, 2014).

The aim of the present work was to better characterize the involvement of PM order and fluidity modifications in early steps of defence signalling using different elicitor–plant cell pairs. By both recording fluorescence properties of the PM labelled with an environment-sensitive probe and following the kinetics of fluorescence recovery after photobleaching (FRAP), we were able to measure PM order and membrane fluidity, when tobacco or Arabidopsis suspension cells were treated by flg22, OG, and cryptogein. Interestingly, we described both similar and divergent PM rearrangements depending on the plant cell–elicitor pairs, and we established the relationship between NADPH oxidase-mediated ROS production and membrane order. Finally, we opened up the discussion of whether the regulation of the PM properties could partially account for the specificity of the signalling pathways and how the common step, namely membrane order control, could be linked to the ‘membrane raft’ model.

## Materials and methods

### Plant material and growth conditions

Wild-type BY-2 (*Nicotiana tabacum* cv. Bright Yellow 2) cells and cells of a BY-2 cell line expressing *NtRbohD* antisense cDNA (gp3) (Simon-Plas *et al.*, 2002) were grown in Murashige and Skoog (MS) modified medium (basal salt mixture, M0221, Duchefa) at pH 5.6, supplemented with 1mg l^−1^ thiamine-HCl, 0.2mg l^−1^ 2,4 dichlorophenylacetic acid, 100mg l^−1^ myo-inositol, 30g l^−1^ sucrose, 200mg l^−1^ KH_2_PO_4_, and 2g l^−1^ MES. Chlorophyllian *A. thaliana* cells (ecotype Columbia) were grown in MS modified medium (including Nitsch vitamins, M0256, Duchefa) at pH 5.7, supplemented with 0.5mg l^−1^naphthalenacetic acid, 50 µg l^−1^ kinetin, and 30g l^−1^ sucrose. Cell suspensions were maintained under continuous light conditions (200 µE m^−2^ s^−1^) on a rotary shaker (140rpm) and diluted weekly (3:80 and 20:100 for tobacco and Arabidopsis, respectively) into fresh medium.

### Reagents

1-[2-Hydroxy-3-(*N*,*N*-di-methyl-*N*-hydroxyethyl)ammoniopropyl]-4-[β-[2-(di-*n*-butylamino)-6-napthyl]vinyl] pyridinium dibromide (di-4-ANEPPDHQ) was purchased from Molecular Probes Inc. Staurosporin, lanthanum (La^3+^), diphenyleneiodonium (DPI), and methyl-β-cyclodextrin (MβCD; Cell Culture Tested) were obtained from Sigma-Aldrich. Hydrogen peroxide (H_2_O_2_, 3 wt % solution in water containing 200ppm acetanilide as stabilizer) was also supplied by Sigma-Aldrich.

### Expression and purification of recombinant proteins

Wild-type cryptogein was purified from *P. cryptogea* culture according to the method of Ricci (1989) and prepared in distilled water (stock solution 0.5mg ml^−1^). Recombinant wild-type cryptogein (Cry X24) and its variants (Cry V84F, Cry L41F, and Cry V84F/L41F) were expressed using the vector pPIC9 with the inserted cryptogein gene (wild type or mutated forms) from *P. cryptogea* with an additional N-terminal glycine residue to improve the processing ability of the KEX2 protease (α-secretion factor cleavage). The constructed vectors were expressed into *Pichia pastoris* strain GS115. Screening for optimal protein production was performed and the most suitable strain was cultivated in a Biostat B-DCU bioreactor (Sartorius) using a previously described protocol (Wood and Komives, 1999). After cultivation, the expressed protein was concentrated by ultrafiltration (cut off 3kDa) and purified by fast protein liquid chromatography using a Source S15 ion-exchange column (GE Healthcare). Molecular weights of isolated proteins were confirmed by MALDI-MS spectroscopy (Supplementary Fig. S1 at *JXB* online). Proteins were quantified by Bradford assay and conserved in distilled water (stock solution 0.5mg ml^−1^).

### Cell treatments

Suspension cells were harvested 7 d after subculture, filtered, and resuspended (0.1g ml^−1^) in an incubation medium (2mM and 10mM MES buffer, for tobacco and Arabidopsis cells, respectively, pH 5.9, containing 175mM mannitol, 0.5mM CaCl_2_, and 0.5mM K_2_SO_4_). After an equilibration period (1h 30 and 3h, for Arabidopsis and tobacco cells, respectively) on a rotary shaker (140rpm) at 25 °C, cells were treated with chemicals as indicated in the figure legends. For elicitation treatments, concentrated (1000-fold) stock solutions in distilled water of lysozyme (from chicken egg white, Sigma-Aldrich), flagellin (flg22 flagellin fragment, AnaSpec, Inc.), OGs [kindly provided by X. Daire (Lecourieux *et al.*, 2005)], and cryptogein were added to cell suspensions at a final concentration of 20nM, 100nM, 50ng ml^−1^, and 50nM, respectively. Nothing was added in untreated conditions. For pharmacological treatments, lanthanum (50 µM, from a stock solution in water), staurosporin (2.5mM, from a stock solution in DMSO), or DPI (5 µM or 20 µM, from a stock solution in DMSO) were added to the cell suspension 5min before elicitation. For the last two treatments, final DMSO concentrations did not exceed 0.5% (v/v) and equivalent volumes of DMSO were added to controls. To achieve sterol depletion, BY-2 suspension cells were incubated for 15min with 5mM MβCD.

### ROS production and extracellular pH modification measurements

ROS production and extracellular pH were measured at intervals, in the incubation medium. The production of H_2_O_2_ was measured by chemiluminescence using luminol and a luminometer (BCL book, Berthold). A 280 µl aliquot of the cell suspension was added to 50 µl of 0.3mM luminol and 300 µl of the assay buffer (175mM mannitol, 0.5mM CaCl_2_, 0.5mM K_2_SO_4_, and 50mM MES pH 6.5). For Arabidopsis cells, 2U of horseradish peroxidase (Sigma-Aldrich) were added to the assay buffer. Extracellular pH modifications were monitored using a pH meter (PHM95, Radiometer).

### Fluorescence labelling

Control and treated suspension cells (500 µl), placed in an eppendorf tube, were labelled with 1 μl of 1.5mM (3 µM final concentration) di-4-ANEPPDHQ stock solution (in DMSO) for 1min, just before analysis.

### Fluorescence spectroscopy

Labelled cells were transferred to a 10mm Special Optic Glass path cuvette. Fluorescence measurements were executed on a Fluorolog-3 FL3-211 spectrometer (Jobin-Yvon, Horiba Group) in the T-format with one double monochromator for excitation and two single monochromators for emission light. Detection was ensured by two photomultipliers. The light source was a xenon arc lamp. The spectrophotometer was equipped with movable excitation and emission polarizers. All fluorescence signals were recorded with emission and excitation bandwidths of 5nm and an integration time of 1s. All data were acquired with the Datamax software (Jobin-Yvon/Thermo Galactic Inc.). Samples were stirred and equilibrated in a temperature-controlled chamber (22 °C) using a thermoelectric Peltier junction (Wavelength Electronics Inc.).

### Confocal fluorescence microscopy

Chlorophyllian cells were observed with a Leica TCS SP2-AOBS laser scanning microscope (Leica Microsystems). Fluorescence excitations were obtained with the 488nm line of an argon laser, and fluorescence emissions were filtered between 545 nn and 565nm and between 635nm and 655nm to record green and red fluorescence intensities, respectively. Images were acquired with a HCPL Apochromat CS ×63 (N.A. 1.40) oil immersion objective to allow for ratiometric processing.

### FRAP experiments

An aliquot of labelled cells was placed between the slide and cover-slip. A Leica TCS SP2-AOBS laser scanning confocal upright microscope (Leica Microsystems) coupled to a 488nm line of an argon laser was used for excitation, with a detection bandwidth of 510–700nm. Cells were observed using a Plan Apo ×40 oil immersion objective (N.A. 1.25) and the detection pinhole was set to the optimum value of 1 Airy unit. All experiments were performed according to Bonneau *et al.* (2010). After five pre-bleach scans (one scan every 800ms) at 8% maximal laser power to determine the initial fluorescence intensity, one photobleaching scan was executed at 100% laser power. Post-bleaching fluorescence recovery was then sampled at 8% laser power for 106s. A second FRAP measurement was systematically performed on the same bleached region.

### Statistical tests

Statistical inference was based on non-parametric tests (Mann–Whitney) since we observed that our data exhibit a non-Gaussian distribution.

## Results

### An increase of PM order is associated with signalling triggered by defence elicitors

**Fig. 1.**
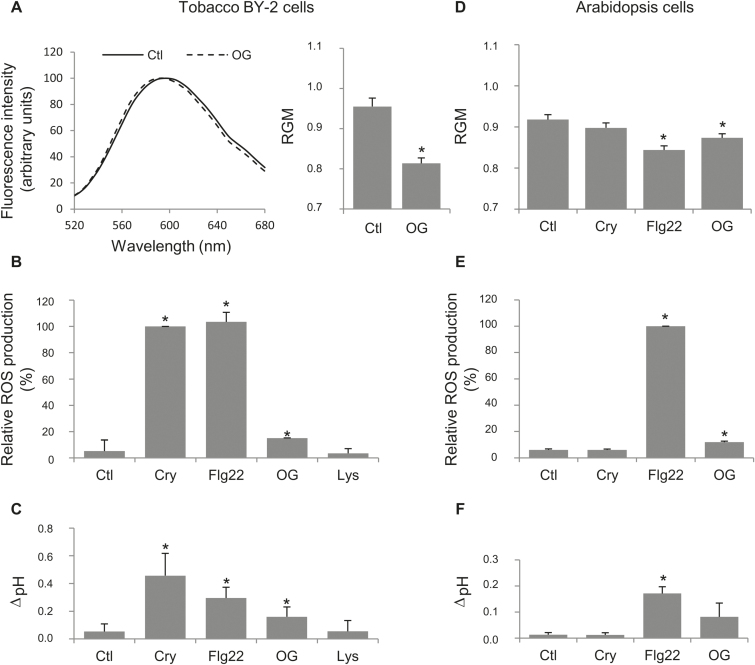
Increase of the PM order is associated with signalling triggered by elicitors of defence. Tobacco (A–C) or Arabidopsis (D–F) cells were exposed to cryptogein (Cry, 50nM), flagellin (Flg22, 20nM), oligogalacturonides (OG, 50ng ml^−1^), or lysozyme (Lys, 100nM) and compared with control cells (Ctl, without treatment). (A, D) After a 5min treatment, cells were labelled with 3 µM di-4-ANEPPDHQ and emission spectra [control (dark line) and elicited (dotted line) cells] were recorded to quantify membrane order modifications using the red/green ratio (RGM, with RGM=I660/I550). (B, E) ROS production was assessed by chemiluminescence and followed during the first 60min after elicitation treatment. The sum of ROS produced during the kinetics was reported relative to the cryptogein-induced (B) or flagellin-induced (E) values (Arbitrary Units). (C, F) Extracellular alkalinization was reported as pH variation after 1h of treatment. Mean values ±SEM (*n*>3 independent experiments). Asterisks highlighted a significant difference compared with the control (*P*-value<0.05).

To assess the effect of different elicitors of defence responses on membrane order, we used di-4-ANEPPDHQ, a membrane fluorescent probe sensitive to local lipid packing (Jin *et al.*, 2006). Modifications of the membrane order revealed by a shift of the di-4-ANEPPDHQ fluorescence emission spectrum are commonly quantified using the ratio of the emission fluorescence intensities recovered at 660nm and 550nm (I660/I550nm) called the RGM, for the red to green ratio of the membrane (Jin *et al.*, 2005; Roche *et al.*, 2008, 2010; Dinic *et al.*, 2011). We previously showed that addition of cryptogein and flg22, elicitors from *P. cryptogea* and *P. syringea*, respectively, increased the PM order in BY-2 cells (Gerbeau-Pissot *et al.*, 2014). To complete the picture of the relationship between such an increase and the defence signalling cascade, we extended the analysis by using another signalling molecule, the elicitor-active OGs. Tobacco suspension cells were treated with OGs (50ng ml^−1^), and subsequently labelled with di-4-ANEPPDHQ (3 µM) 1min before emission fluorescence recording. We observed, after 5min of OG elicitation, a blue shift of the emission spectrum and a concomitant decrease of RGM value (Fig. 1A), characteristic of an increase of PM order. All elicitors identified as competent to modify RGM, namely cryptogein (50nM), flg22 (20nM), and OG (Supplementary Fig. S2) in tobacco BY-2 cells, are also able to induce two typical early defence events—ROS production (Fig. 1B) and pH alkalinization (Fig. 1C). In contrast, lysozyme (100nM), an inactive protein, did not trigger either a significant change in tobacco PM order (Supplemental Fig. S2) or signalling events during the time course of the experiment (Fig. 1), suggesting a strong correlation between induction of a signalling cascade and promotion of a membrane order increase by elicitation.

To address the plant specificity of this mechanism, we extended the analysis to another common elicitor–plant cell pair, using Arabidopsis cells and the flg22 peptide, which is a potent elicitor of the defence response in this plant (Gomez-Gomez and Boller, 2002). Flg22 elicitation triggered on Arabidopsis cells a significant decrease of RGM value within 5min of treatment (Fig. 1D), and also induced both a rapid ROS production and an extracellular alkalinization (Fig. 1E, F), endorsing the relationship between the RGM decrease and signalling cascade triggering by elicitors. In agreement with this, cryptogein, which is not able to induce a signalling cascade in Arabidopsis cells (Bourque *et al.*, 1999), provoked neither RGM modification nor ROS production and pH alkalinization (Fig. 1D–F), whereas OG, which was proved to be able to induce an oxidative burst in Arabidopsis cells (Galletti *et al.*, 2008), was also able to decrease RGM in these cells (Fig. 1D).

Altogether, these data indicate that all elicitors tested were able to induce a rapid increase of membrane order, whenever early markers of defence signalling, notably ROS production, were observed. Moreover, such membrane modification is induced by elicitors in different plant cells, suggesting that it could correspond to a generic early event of the elicitor-triggered signalling cascade.

**Fig. 2.**
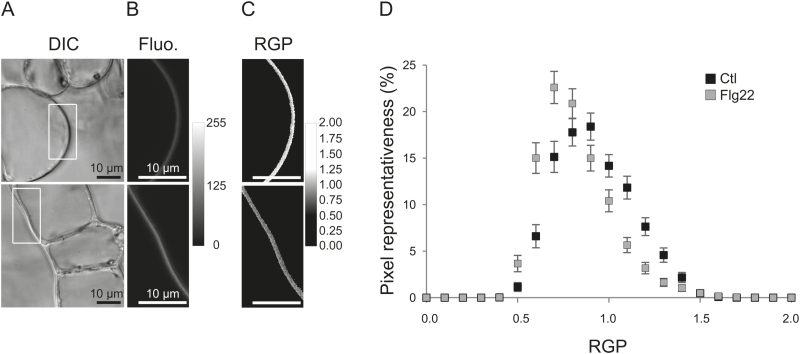
Increase of the representativeness of PM ordered domains by flg22 in *Arabidopsis thaliana* cells. Arabidopsis cells were observed after elicitation treatment with 20nM flg22 (bottom) or in control conditions (top). (A) Differential interference contrast (DIC) microscopy images. (B) Fluorescence (Fluo.) of plasma membrane of di-4-ANEPPDHQ-labelled Arabidopsis cells (excitation at 488nm; emission corresponds to the sum of fluorescence intensities acquired from channels ranging from 520nm to 680nm) with a grey scale rendering of fluorescence intensity. (C) Ratiometric images of di-4-ANEPPDHQ- (3 µM) labelled Arabidopsis cells were pseudocolour-coded, according to the accompanying RGP value scale showing the membrane order. A zoom of an area extracted from the PM surface is displayed. Scale bar=10 µm. (D) Each pixel constituting the analysed PM surface was associated with its own level of order (RGP) and a comparison of the distribution of the membrane order between flagellin-treated (Flg22, grey squares) and control cells (Ctl, black squares) is given. The *x*-axis represents the class of order level values. The *y*-axis represents the percentage of each class of pixel values. Data are means ±SE of the mean (*n*=206 cells, from four independent experiments).

The increase of PM order has been previously associated in tobacco cells elicited with cryptogein with an increase of the relative proportion of ordered domains (Gerbeau-Pissot *et al.*, 2014). To determine if a similar reorganization of PM lateral compartmentalization is involved in other elicitation treatments, the di-4-ANEPPDHQ emission spectrum was analysed for small regions of interest (ROIs, 300×300nm square) in the PM of flg22-treated Arabidopsis cells (Fig. 2A–C). Interestingly, after 5min of flg22 treatment, an enrichment of ROIs exhibiting a higher membrane order was observed at the Arabidopsis PM surface (Fig. 2D), showing that the increase of global PM order is related to an increase of the representativeness of ordered domains. Since comparable effects are observed for both cryptogein–tobacco and flg22–Arabidopsis pairs, these new data emphasize a generic mechanism of elicitor-induced regulation of PM order.

### Elicitor-induced increase of PM order depends on Ntrboh-mediated ROS production

**Fig. 3.**
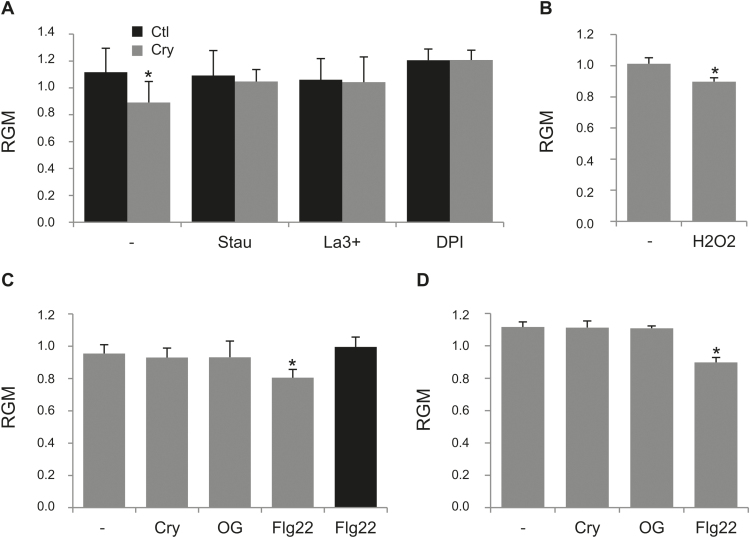
Elicitor-induced increase of PM order depends on ROS production. (A) Effect of signalling inhibitors (staurosporin, Stau, 2.5mM; lanthanum, La^3+^, 50 µM; diphenyleneiodonium, DPI, 5 µM; none, –) on RGM of di-4-ANEPPDHQ-labelled tobacco BY-2 cells without elicitor treatment (Ctl, black histograms) or after a 5min cryptogein elicitation (Cry, 50nM, grey histograms). (B) Effect of H_2_O_2_ addition (100 µM) on RGM of di-4-ANEPPDHQ-labelled tobacco BY-2 cells. (C) Effect of the NADPH oxidase activity inhibitor DPI (5 µM, grey histograms, or 20 µM, black histogram) on RGM of di-4-ANEPPDHQ-labelled tobacco BY-2 cells treated with different elicitors (–, none; Cry, 50nM; OG, 50ng ml^−1^; or Flg22, 20nM). (D) Effect of elicitors (–, none; Cry, 50nM; OG, 50ng ml^−1^; or Flg22, 20nM) on RGM of di-4-ANEPPDHQ-labelled tobacco gp3 cells. Mean values ±SEM (*n*>4 independent experiments). Asterisks indicate a significant difference (*P*-value <0.05).

To determine further the link between the increase in PM order and the signalling cascade triggered by elicitors, we used selective inhibitors of early signalling events. It has been reported that the cryptogein response of tobacco BY-2 cells involved rapid protein phosphorylations essential for transduction signals (Viard *et al.*, 1994; Lebrun-Garcia *et al.*, 1999), including variations in free calcium concentration (Lecourieux *et al.*, 2002) and ROS production (Simon-Plas *et al.*, 2002). Consistently, addition of staurosporin (2.5mM) 5min before cryptogein elicitation completely abolished the elicitor-induced signalling pathway (Viard *et al.*, 1994). Here, staurosporin also prevented the RGM decrease observed 5min after cryptogein treatment (Fig. 3A), suggesting that this membrane modification may be regulated either directly by phosphorylation or by a downstream cascade dependent on phosphorylations. We further tested other inhibitors hindering the subsequent events involved in cryptogein signalling: lanthanum for calcium influx (50 µM, 5min) and DPI for ROS production (5 µM, 5min). Both treatments abolished the cryptogein-induced RGM decrease (Fig. 3A), indicating that modification of the PM order is linked to these signalling events.

As NADPH oxidases, such as NtRbohD responsible for the oxidative burst triggered by cryptogein (Simon-Plas *et al.*, 2002), are regulated by calcium and phosphorylations (Kobayashi *et al.*, 2007; Ogasawara *et al.*, 2008), our results support a possible close link between RGM decrease and ROS production. Elicitation-induced oxidative burst includes production of unstable superoxide (O_2_·^–^) rapidly converted into H_2_O_2_. When H_2_O_2_ (100 µM) was added to tobacco BY-2 cells, a significant decrease of RGM observed after 5min treatment (Fig. 3B) confirms the ability of this ROS to modulate membrane order.

The relationship between membrane order increase and ROS production triggered by elicitors was further investigated by measuring the effect of DPI addition on the RGM decrease induced by other elicitors (Fig. 3C). In cells treated with OGs, DPI addition inhibited ROS production (Supplementary Fig. S3) and prevented RGM decrease (Fig. 3C). Since DPI is a well known inhibitor of flavocytochromes, this result infers that membrane order modification depends on NADPH oxidase-mediated ROS production, in agreement with previously identified involvement of RBOH enzymes. Using the same concentration of DPI (5 µM), a very low residual ROS production was still observed in flg22-treated BY-2 cells (Supplementary Fig. S3), together with a significant decrease of RGM (Fig. 3C), whereas a higher concentration of the inhibitor (20 µM) completely inhibited both ROS production and RGM decrease (Fig. 3C; Supplementary Fig. S3). This finding may support the participation of several NADPH oxidases with different sensitivity to DPI in the signalling pathways induced by various elicitors. To strengthen the dependence of membrane order on ROS production, we further analysed RGM modifications in tobacco gp3 cells (a BY-2 cell line expressing *NtRbohD* antisense cDNA; Simon-Plas *et al.*, 2002) unable to produce ROS when treated with cryptogein (Supplementary Fig. S4A). No modification of PM order was observed when OGs or cryptogein were added to gp3 cells (Fig. 3D), indicating that both elicitors are unable to reduce RGM in the absence of *NtRbohD*-mediated ROS production (Supplementary Fig. S4B), while H_2_O_2_ addition (100 µM) induced a decrease in RGM (Supplementary Fig. S4C) similar to that of BY-2 cells. Consistent with results obtained in the presence of DPI, flg22 treatment triggered in gp3 suspension cells a significant increase of ROS production (Supplementary Fig. S4B) together with a decrease of RGM (Fig. 3D), confirming the activation by this elicitor of an isoform of NADPH oxidase other than NtRbohD.

Altogether, these data clearly indicate that an RGM decrease is a part of the generic signalling cascade induced by different elicitors, and is dependent on very early ROS production (already detectable 5min after treatment; Supplementary Fig. S5) mediated by NADPH oxidases, irrespective of the RBOH isoform involved.

### Enhancement of PM fluidity by cryptogein is linked to its sterol-trapping activity

**Fig. 4.**
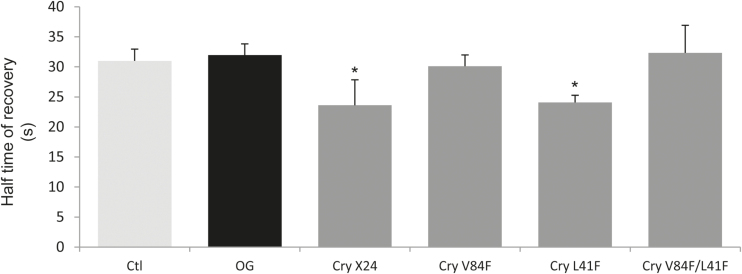
Effect of defence elicitors on PM fluidity of BY-2 cells. Tobacco BY-2 cells were treated with either oligogalacturonides (OG, black histogram) or cryptogein variants affected in their ability to load up sterols (grey histograms). After a 5min treatment, cells are labelled with di-4-ANEPPDHQ (3 µM) and half-time recovery of maximal fluorescence after photobleaching was measured for untreated (Ctl, white histogram) or elicited cells (50ng ml^−1^ OG, or 50nM of different purified cryptogein variants: Cry X24 corresponding to the wild type form of cryptogein produced in *Pichia pastoris*; Cry V84F, Cry L41F, and Cry V84F/L41F corresponding to mutated forms). Mean values ±SEM (*n*>4 independent experiments). Asterisks denote a statistically significant difference (*P*-value <0.05).

In contrast to the common elicitation-induced increase of membrane order, previous results comparing cryptogein- and flg22-induced effects on membrane fluidity indicated that only cryptogein was effective in modulating membrane lateral fluidity (Gerbeau-Pissot *et al.*, 2014). To confirm such specificity of cryptogein elicitation, we evaluated the effect of OG elicitation on fluidity, through the lateral diffusion of the di-4-ANEPPDHQ fluorescent probe within the PM measured by FRAP experiments. After labelling of BY-2 cells, the PM was submitted to photobleaching and the dye mobility was followed by the recovery of fluorescence. After 5min of OG elicitation, BY-2 cells exhibited the same fluorescence recovery kinetics as control cells, with a half-time of fluorescence recovery (*t*_1/2_) of 31.6s (± 1.4, *n*=58 cells) and 33.1s (± 1.3, *n*=34 cells) for control and elicited cells, respectively (Fig. 4), associated with the same mobile fraction (Supplementary Fig. S6). Conversely, after 5min of incubation, cryptogein, used as a positive control, was able to induce a significant modification to the lateral fluidity of these cells (Fig. 4). The present data demonstrate that OG treatment does not influence PM fluidity and reinforce the hypothesis of a specific ability of cryptogein to affect this parameter.

A specific characteristic feature of cryptogein is its capacity to load up sterols efficiently from biological or artificial membranes into a hydrophobic pocket (Mikes *et al.*, 1998; Vauthrin *et al.*, 1999; Osman *et al.*, 2001). To investigate possible links between modification of PM fluidity and sterol-trapping activity induced by cryptogein, BY-2 cells were elicited with cryptogein variants exhibiting differential sterol-trapping capacities (Fig. 4). The recombinant wild-type cryptogein (X24) was compared with the variants Val84Phe (Cry V84F) and Leu41Phe (Cry L41F) carrying a single mutation, and the corresponding double mutant (Cry V84F/L41F). The mutations target the hydrophobic cavity of the protein without any physico-chemical changes notably in the ω-loop conformation or overall structure of the proteins (Supplementary Fig. S1), and alter both binding and trapping of lipid compounds (Dokladal *et al.*, 2012). When BY-2 cells were treated with 50nM Cry V84F having a significantly reduced ability to trap sterols from membranes (Dokladal *et al.*, 2012), no stimulation of PM fluidity was observed (Fig. 4). On the other hand, the same concentration of the Cry L41F mutant, affected in fatty acid but not in sterol binding activity (Dokladal *et al.*, 2012), increased PM fluidity as did the wild-type X24 cryptogein (Fig. 4). In addition, the double mutant Cry V84F/L41F, having no detectable sterol-trapping activity, was not able to increase PM fluidity (Fig. 4).

These results strongly connect the specific ability of cryptogein to increased PM fluidity with its sterol binding abilities.

### Cryptogein-induced increase of PM fluidity is not dependent on early signalling events but could enhance ROS production intensity

**Fig. 5.**
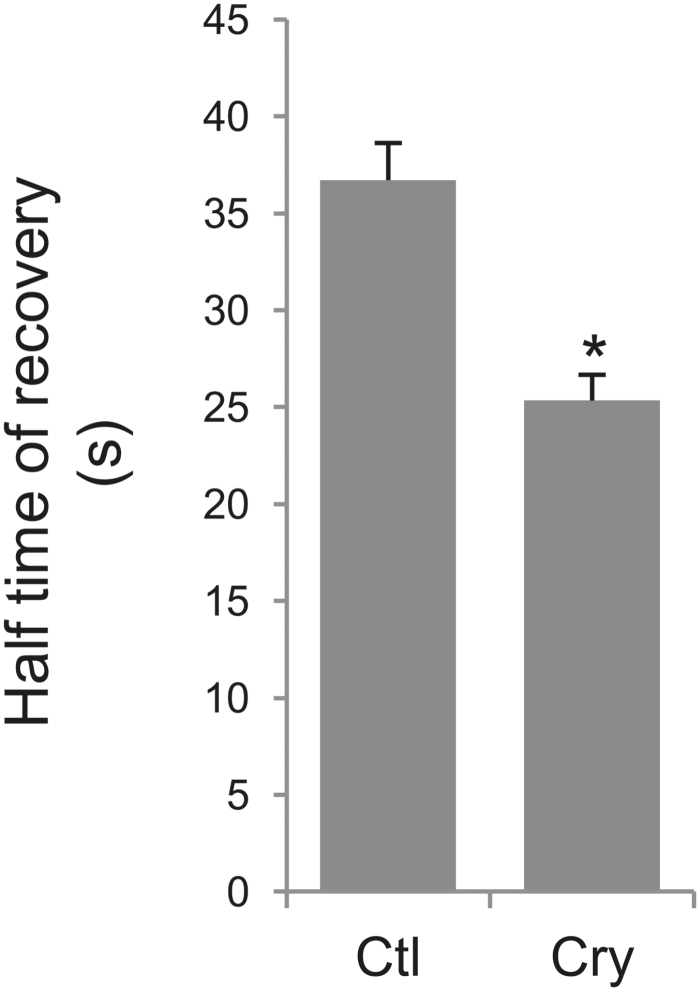
Cryptogein induced an increase of PM fluidity in Arabidopsis cells. FRAP experiments were performed on di-4-ANEPPDHQ-labelled Arabidopsis suspension cells after a 5min treatment, and half-times of fluorescence recovery were reported. Control corresponds to no addition of elicitor, 5min. Mean values ±SEM (*n*>3 independent experiments) and an asterisk denotes a statistically significant difference (*P*-value <0.05).

To identify a possible link between the increase of membrane fluidity and the signalling cascade triggered by cryptogein, FRAP experiments were performed on another plant cell–elicitor pair. FRAP experiments performed on *A. thaliana* suspension cells, which do not undergo any detectable signalling event upon cryptogein treatment, including ROS production (Fig. 1E), showed a significant decrease of the *t*_1/2_, from 36.7s (± 1.9, *n*=28 cells) for control cells to 25.3s (± 1.3, *n*=28 cells) for cells treated with the wild-type X24 cryptogein (Fig. 5), indicating an increase of PM fluidity without requiring induction of a signalling cascade.

**Fig. 6.**
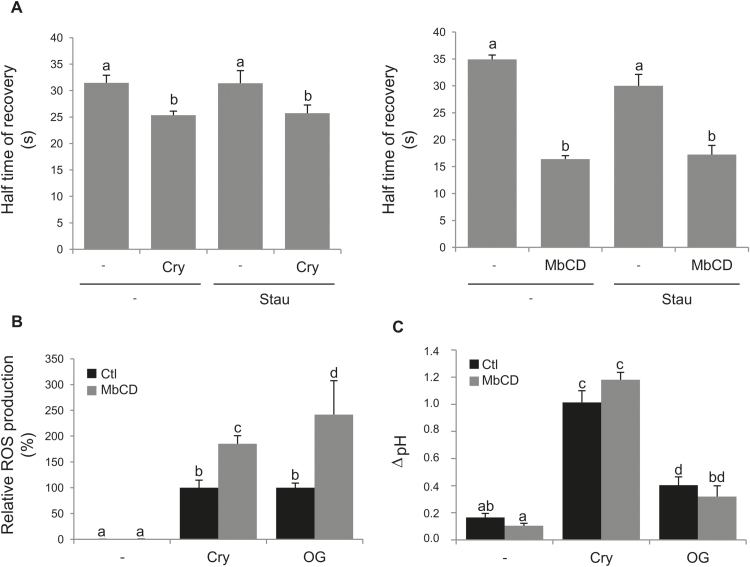
Sterol trapping enhances ROS production induced by the elicitation signalling cascade. (A) Fluidity is enhanced by sterol depletion. Half-maximal time of fluorescence recovery was measured by FRAP experiments after sterol depletion (5min of a 50nM cryptogein elicitation, cry, or 15min of a 5mM methyl-β-cyclodextrin treatment, MβCD) and/or phosphorylation inhibition (by a 5min incubation with 2.5mM staurosporin, Stau). (B and C) Early events of the signalling cascade were measured in control conditions (no addition in the medium, –) and after elicitation treatment with either 50nM cryptogein (Cry) or 50ng ml^−1^ oligogalacturonides (OG). A pre-incubation with (grey histogram) or without (black histogram) 5mM MβCD was performed to evaluate the effect of sterol trapping on these parameters. (B) ROS production measurement. The sum of the ROS production during the first 60min was measured by chemiluminescence and reported relative to the elicitor-induced treatment value (without sterol depletion). (C) pH alkalinization was evaluated after 30min of MβCD treatment. Mean values ±SEM (*n*>5 experiments). Letters indicate a significant difference between treatments (*P*-value <0.05).

According to these results, addition of the kinase inhibitor staurosporin (2.5mM) to BY-2 cells 5min before cryptogein treatment did not significantly modify the elicitor-induced decrease of half-time fluorescence recovery (Fig. 6A), implying that the increase of PM fluidity induced by cryptogein is neither directly regulated by early phosphorylation events, nor dependent on the subsequent steps of the cryptogein-induced signalling cascade. Altogether, these results indicate that cryptogein exhibits a specific ability to modulate PM fluidity, independently of the activation of a signalling cascade.

To explore further a possible effect of such a cryptogein-induced increase of PM fluidity on defence signalling, we used MβCD (5mM, 15min), a cyclic oligosaccharide able to deplete sterols from the PM of living cells (Christian *et al.*, 1999). The sterol-trapping activity of MβCD is similar to that of cryptogein, whereby they both do not require any energy transfer and only result from the high affinity of the two molecules for sterols (Lopez *et al.*, 2013). A significant increase of the PM fluidity of BY-2 cells, with a 1.6-fold decrease of the t_1/2_, was observed after MβCD treatment (Fig. 6A), without change in mobile fractions (Supplementary Fig. S7). Staurosporin did not inhibit the MβCD-induced increase of PM fluidity, as for the cryptogein-induced increase of PM fluidity (Fig. 6A). It is noteworthy that MβCD alone was not able, in our experimental conditions, to induce any detectable signalling events such as ROS production (Fig. 6B) and pH alkalinization (Fig. 6C). However, when added 15min before treatment of BY-2 cells by either cryptogein (50nM) or OG (50ng ml^−1^), MβCD induced a significant increase of ROS production compared with the elicitors alone (Fig. 6B), whereas the extracellular alkalinization was not affected (Fig 6C). These data suggest that MβCD treatment that is able to increase fluidity through sterol trapping enhances elicitor-induced ROS production, whatever the biotic agent used. Interestingly, compounds commonly used to increase PM fluidity (Mrak, 1992; Blixt *et al.*, 1993) without modifying the amount of sterol, namely benzyl alcohol (BA; 20mM) and ethanol (0.1%), were not able to enhance cryptogein-induced ROS production (Supplementary Fig. S8). Our result thus demonstrates that an increase in membrane fluidity resulting from the mechanical trapping of sterols from the PM is able to increase the intensity of ROS production triggered by defence elicitors.

**Fig. 7.**
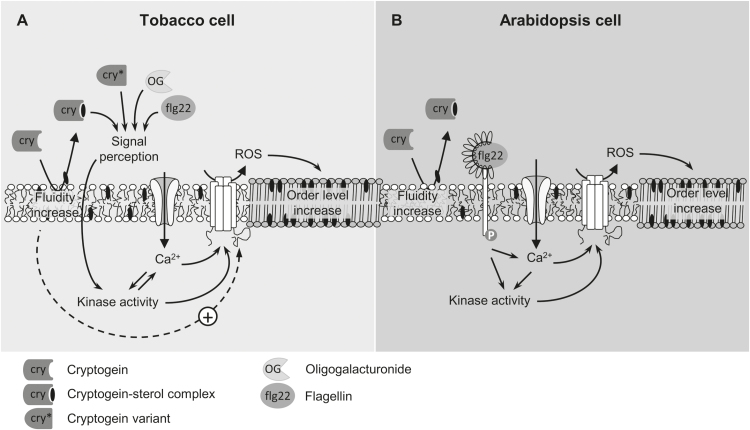
Membrane events of the early signalling cascade induced in plant suspension cells by defence elicitors. (A) In tobacco cells, a different mode of recognition could be proposed to detect cryptogein (cry), flagellin (flg22), and oligogalacturonide (OG). Elicitor perception triggers kinase activation that leads to calcium influx. Both mechanisms are able to activate the NADPH oxidase-driven ROS burst, which in turn increases membrane order. In parallel, the sterol-trapping ability of cryptogein leads to an increase in membrane fluidity which enhances the ROS burst intensity. (B) In Arabidopsis cells, upon flg22 binding to its receptor, complex formation triggers rapid phosphorylation events. The signal transduction downstream of ligand perception includes a Ca^2+^ burst and activation of RBOH required for the ROS burst. Activation of RBOH is required for the increase of membrane order. In this cell context, cryptogein is still able to trap sterol, and concomitantly to increase membrane fluidity, without inducing any signalling pathway.

Therefore, our results raise the possibility that elicitors of plant defence are able to modify membrane order in a ROS-dependent manner, whereas only cryptogein would be able, through its sterol-trapping activity, to increase membrane fluidity, thus stimulating ROS production (Fig. 7).

## Discussion

### Membrane order increase is a novel player in signalling cascades initiated by defence elicitors

In addition to conserved basal defence responses, membrane order increase was established in diverse immunity systems. Indeed, in lymphocytes, it has been demonstrated that a higher membrane order resides at the immunological synapse periphery where the signalling cascade takes place (Gaus *et al.*, 2005), and that such a patterning of membrane order depends on active signalling (Owen *et al.*, 2010). In the present study, we described an increase of membrane order during the first minutes after treatment of tobacco or Arabidopsis suspension cells with an elicitor of the defence reaction regardless of the nature of the elicitor, provided that an active signalling process is engaged. Membrane rearrangement might thus be the hallmark of the early defence signalling process across different kingdoms.

Furthermore, stimulation of T lymphocytes has been shown to induce redistribution and clustering of ordered domains at the site of T-cell receptor engagements (Viola *et al.*, 1999). Anisotropy measurements also permitted the demonstration that the recruitment of lipid molecules into more ordered domains that serve as platforms for IgE-mediated signalling (Davey *et al.*, 2007) provides a general mechanism for amplifying signalling by reorganization of membrane ordered domains. In perfect line with this concept is the ‘membrane raft’ hypothesis assuming the ability of areas with different biophysical properties (Simons and Ikonen, 1997) to coalesce into larger structures, particularly in response to pathogens (Lingwood and Simons, 2010; Simons and Sampaio, 2011). Accordingly, we demonstrated that the increase of global membrane order is associated with an increase in the relative proportion of the most ordered domains, in the case of both cryptogein/tobacco cells (Gerbeau-Pissot *et al.*, 2014) and flg22/Arabidopsis cells, indicating that similar mechanisms of signalling platform formation might occur within the first steps of both animal and plant defence. Thus, both membrane raft formation (Gerbeau-Pissot *et al.*, 2014) and the concentration of key players in membrane rafts (Stanislas *et al.*, 2009; Keinath *et al.*, 2011) have been evidenced during the immune response in plant cells.

### NADPH oxidase-mediated ROS production could account for the increase in membrane order

Along the signalling cascade, generation of ROS has been proved to be mediated notably through the activity of PM-associated NADPH oxidases of the RBOH family (Marino *et al.*, 2011), particularly the D isoform in tobacco (Simon-Plas *et al.*, 2002). Involvement of NtRbohF, associated with the defence response in other immune systems (Galletti *et al.*, 2008; Pogany *et al.*, 2009; Asai and Yoshioka, 2009; Zhang *et al.*, 2009), could be proposed from our result in the flg22-induced signalling pathway in tobacco cells, where the role of NtRbohD was proved in cryptogein- and OG-induced membrane order increase. In line with such specific roles of RBOH isoforms in plant immunity, differential spatio-temporal expression patterns have been defined for *AtRbohD* and *AtRbohF* genes during the Arabidopsis immune response to both hemibiotrophic bacteria and necrotrophic fungal pathogens (Morales *et al.*, 2016). Moreover, the concomitant detection of oxidative burst and increase of membrane order a few minutes after elicitor treatment indicates a close proximity between the localization of RBOH proteins and players involved in membrane order modifications, namely ordered domains. This hypothesis is strongly supported by (i) the sensitivity to DPI treatment; (ii) the exclusive association of NtRbohD with the detergent-resistant membrane (DRM) fraction (Morel *et al.*, 2006; Roche *et al.*, 2008); (iii) the clustered distribution of NtRbohD at the PM surface (Noirot *et al.*, 2014); and (iv) the discrete distribution along the PM, within patches of ~80nm, of H_2_O_2_ derived from this enzyme activity 5min after cryptogein treatment (Lherminier *et al.*, 2009).

Although ROS production and now an increase in membrane order could be considered as generic players involved in the signalling cascade to different elicitors, such as cryptogein, flagellin, and OGs, some discrepancies concerning the intensity of the response triggered by these different elicitors have been underlined in our work. However, it should be noted that an increase in membrane order is always induced whatever the ROS production intensity. Such an absence of a dose–response relationship argues in favour of a trigger role for ROS in the signalling cascade leading to membrane reorganization. In agreement with this, ROS could act at different steps of the cell defence response, including regulation of signal transduction (Barna *et al.*, 2012). ROS involvement in regulation of gene expression (Galletti *et al.*, 2008; Kulik *et al.*, 2015) or in cell death induction (Torres, 2010; Wang *et al.*, 2013) has also been proposed. Furthermore, ROS, when produced locally at low concentration, can act as second messengers (Chan *et al.*, 2015), including regulation of PM components recycling (Yan *et al.*, 2015). In plant cells, H_2_O_2_ was proposed to govern the subcellular redistribution of PM aquaporin (Wudick *et al.*, 2015) and to control the cryptogein-induced endocytosis (Leborgne-Castel *et al.*, 2008). We now have to determine in which of these pathways governed by ROS production membrane order is involved.

We proposed that RBOH-dependent ROS production increases PM order by stimulating the formation of ordered domains at the PM surface, as described in the ‘membrane raft’ hypothesis (Lingwood and Simons, 2010). Taking into account the very rapid regulation process potentially involved, modifications of lipid packing in response to oxidative damage could be proposed, as observed in response to tert-butyl hydroperoxide (Borchman *et al.*, 1992). Indeed, oxidized derivatives of sterols have been established as raft promoters (Wang *et al.*, 2004). Moreover, oxidized phospholipid isomers produced by non-enzymatic peroxidation via ROS, notably hydroxyl radicals generated by oxidases such as NADPH oxidases, have been demonstrated to induce both an increase of lipid packing density (Howland and Parikh, 2010) and a more rigid organization related to cross-linking between lipid–lipid moieties (Eichenberger *et al.*, 1982; Bruch and Thayer, 1983; Schroeder, 1984). On the other hand, overproduction of membrane protein carbonylation, another marker of oxidative damage, could explain, to some extent, a viscosity increase in erythrocyte membranes after an acute period of exercise (Berzosa *et al.*, 2011). Such involvement of lipid and/or protein modifications in the increase in membrane order could be sought in our system, since the low level of ROS required to activate membrane adjustment argues in favour of a very specific, localized mechanism. Moreover, the regulation of immune responses by lipid peroxidation observed in plant cells (Polkowska-Kowalczyk and Maciejewska, 2001; Amari *et al.*, 2007) reinforces our assumption.

### Specific cryptogein ability to induce PM fluidity highlights discrepancies between the different defence signalling cascades

Interestingly, cryptogein is the only elicitor, among those tested in this study, able to enhance membrane fluidity. Cryptogein belongs to the family of elicitins, characterized by their singular ability to trap sterol from the PM, this feature being crucial in the signalling process (Osman *et al.*, 2001; Hirasawa *et al.*, 2004). At the same time, the ability of phytosterols to maintain membranes in a microfluid state, important for biological processes, has been well described (Halling and Slotte, 2004; Roche *et al.*, 2008; Gerbeau-Pissot *et al.*, 2014; Grosjean *et al.*, 2015). Correlating cryptogein ability to trap sterol from the PM and its capacity consequently to modify PM fluidity is thus tantalizing. Such a hypothesis was demonstrated using both cryptogein variants with altered ability to bind sterols and MβCD.

Although cyclodextrins could be evoked as inducers of plant defence responses (Morales *et al.*, 1998; Almagro *et al.*, 2012), this facet strongly depends on the derivative used (Bru *et al.*, 2006; Valitova *et al.*, 2014). No signalling cascade, assessed by ROS production or extracellular alkalinization, was engaged in our study using MβCD. A clear fluidization of the cell PM was induced by this compound, and replicated by cryptogein, signifying that a simple sterol trapping is unable to induce a signalling cascade. However, the increase of PM fluidity, dependent on sterol-trapping activity, might act as an enhancing factor of elicitor-induced ROS production, as observed with MβCD in this study and in agreement with the synergistic effect obtained with a combination of cyclodextrin and the potent elicitor methyl jasmonate (Lijavetzky *et al.*, 2008). Thus, our data hint at a model assuming two roles for cryptogein: as a signalling cascade inducer including modification of membrane order; and as a ROS production enhancer through sterol trapping from the PM, and thus increasing PM fluidity (Fig. 7). In agreement with this, several signal transduction pathways leading to cell death, oxidative burst, and expression of defence genes have been proposed and demonstrated to be branched in the early stages following elicitin recognition by tobacco cells (Sasabe *et al.*, 2000). Such a co-operative effect could explain how cryptogein would exhibit a strong ability to induce defence responses. Indeed, cryptogein provokes hypersensitive-related necrosis in tobacco plants (Ricci *et al.*, 1989) and cell death in tobacco cells (Hirasawa *et al.*, 2004; Kadota *et al.*, 2004), whereas flg22 and OGs trigger a signalling cascade without inducing cell death.

Our work went further in the deciphering of the early steps of plant defence signalling, revealing both the modulation of PM order as a new generic player and the key role of the oxidative burst in control of such a PM biophysical property. In parallel, the specific ability of cryptogein to increase PM fluidity, mediated by its sterol-trapping activity, brings new insight into means by which PM organization might be differently involved in defence signalling pathways. Our results pave the way to elucidate both the molecular mechanisms underlying regulation of PM physical parameters and the precise role of such modification in the immune response of plant cells.

## Supplementary data

Supplementary data are available at *JXB* online.

Figure S1. Characterization of cryptogein variants.

Figure S2. Characterization of membrane order modification occurring in elicited cells.

Figure S3. Elicitation-induced ROS production is dependent on NADPH oxidase activity in BY-2 cells.

Figure S4. Relationship between ROS production and PM order in gp3 cell lines.

Figure S5. Kinetics of ROS production induced by cryptogein in tobacco cells.

Figure S6. Effect of oligogalacturonides (OGs) on PM fluidity of BY-2 cells.

Figure S7. Influence of sterol depletion on the mobile fraction during two consecutive bleach and re-bleach sessions.

Figure S8. Fluidizers without sterol-trapping capacity are not able to enhance the oxidative burst.

Supplementary Data

## Abbreviations:

DAMP: damage-associated molecular pattern
MβCD: methyl-β-cyclodextrin;
M/PAMP: microbe/pathogen-associated molecular pattern
OG: oligogalacturonide
PM: plasma membrane
RBOH: respiratory burst oxidase homologue
RGM: red to green ratio of the membrane
ROS: reactive oxygen species.
