# Supplementary material for: Plasma membrane order and fluidity are diversely triggered by elicitors of plant defence
Source: J Exp Bot. 2016 Jul 18;67(17):5173–85. doi: 10.1093/jxb/erw284 (PMC5014163; doi:10.1093/jxb/erw284)
Supplement: Supplementary Data [file supp_67_17_5173__index.html]

Plasma membrane order and fluidity are diversely triggered by elicitors of plant defence — Plasma membrane order and fluidity are diversely triggered by elicitors of plant defence — Supplementary Data 

# Plasma membrane order and fluidity are diversely triggered by elicitors of plant defence

## Supplementary Data

Data files

- supplementary\_figures\_S1\_S8.pdf - Supplementary Data
