# Supplementary material for: Plasma membrane order and fluidity are diversely triggered by elicitors of plant defence
Source: J Exp Bot. 2016 Jul 18;67(17):5173–85. doi: 10.1093/jxb/erw284 (PMC5014163; doi:10.1093/jxb/erw284)
Supplement: Supplementary Data [file supp_erw284_supplementary_figures_S1_S8.pdf]

A

	Maldi MS found masses (Da)
Cry X24	10°386
Cry V84F	10°433
CryL41F	10°418
Cry V84F/L41F	10°467

B

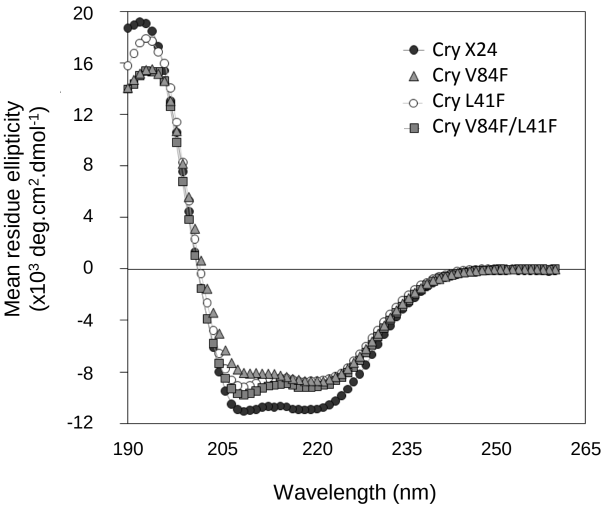

Fig. S1. Characterization of cryptogein variants. (A) MALDI-MS and MS/MS analyses were performed on an Ultraflex III mass spectrometer (Bruker Daltonik, Bremen, Germany). Peptide maps were acquired in reflectron positive mode (25 kV acceleration voltage) with 800 laser shots. Twelve dominant peaks within 700–3600 Da mass range and minimum S/N 10 were picked out for MS/MS analysis employing LID-LIFT arrangement with 600 laser shots for each peptide. CHCA solution prepared according to Havlis *et al.* (2003) was used as the matrix in combination with AnchorChip target to enhance measurement sensitivity. Sample (1 ml) was mixed with matrix solution on the target in a 2:1 ratio. Known autoproteolytic products of trypsin were used for internal calibration of digested peptides. In the absence of these products, an external calibration procedure was employed, using a mixture of seven peptide standards (Bruker Daltonik) covering the mass range of 1000–3100 Da. The Flex Analysis 3.0 and MS Biotools 3.1 (Bruker Daltonik) software were used for data processing. MALDI MS founded masses were reported in a table. The observed mass difference between measured and theoretical MWs (6 Da) corresponded to three disulphide bridges. (B) The proteins overall structure was confirmed by circular dichroism spectroscopy.

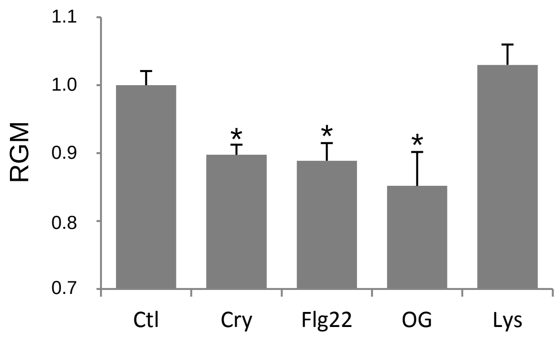

Fig. S2. Characterization of membrane order modification occurring in elicited cells. Tobacco cells were exposed to cryptogein (Cry, 50 nM), flagellin (Flg22, 20 nM), oligogalacturonides (OG, 50 ng.ml^{-1}), or lysozyme (Lys, 100 nM) and compared to control cells (Ctl, without treatment). After a 5 min treatment, cells were labelled with 3 µM of di-4-ANEPPDHQ and membrane order level modifications were quantified using the Red/Green ratio (RGM, with RGM = I660/I550). Mean values ± SD (n>5 independent experiments). Asterisks highlighted a significant difference compared to the control (p value<0.05).

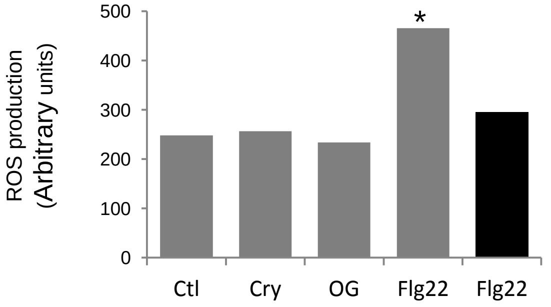

Fig. S3. Elicitation-induced ROS production is dependent on NADPH oxidase activity in BY-2 cells. Effect of elicitors (Cry, 50 nM; OG, 50 ng.ml^{-1} or Flg22, 20 nM) on ROS production of BY-2 cells was measured by chemiluminescence in presence of NADPH oxidase activity inhibitor DPI (5 µM, grey histograms, or 20 µM, black histogram), added 5 min before elicitation treatment. ROS production corresponding to the ROS produced as soon as the 5 first min of treatment was reported. One representative experiment (n>4 independent experiments). Asterisks indicate a significant difference (p value<0.05).

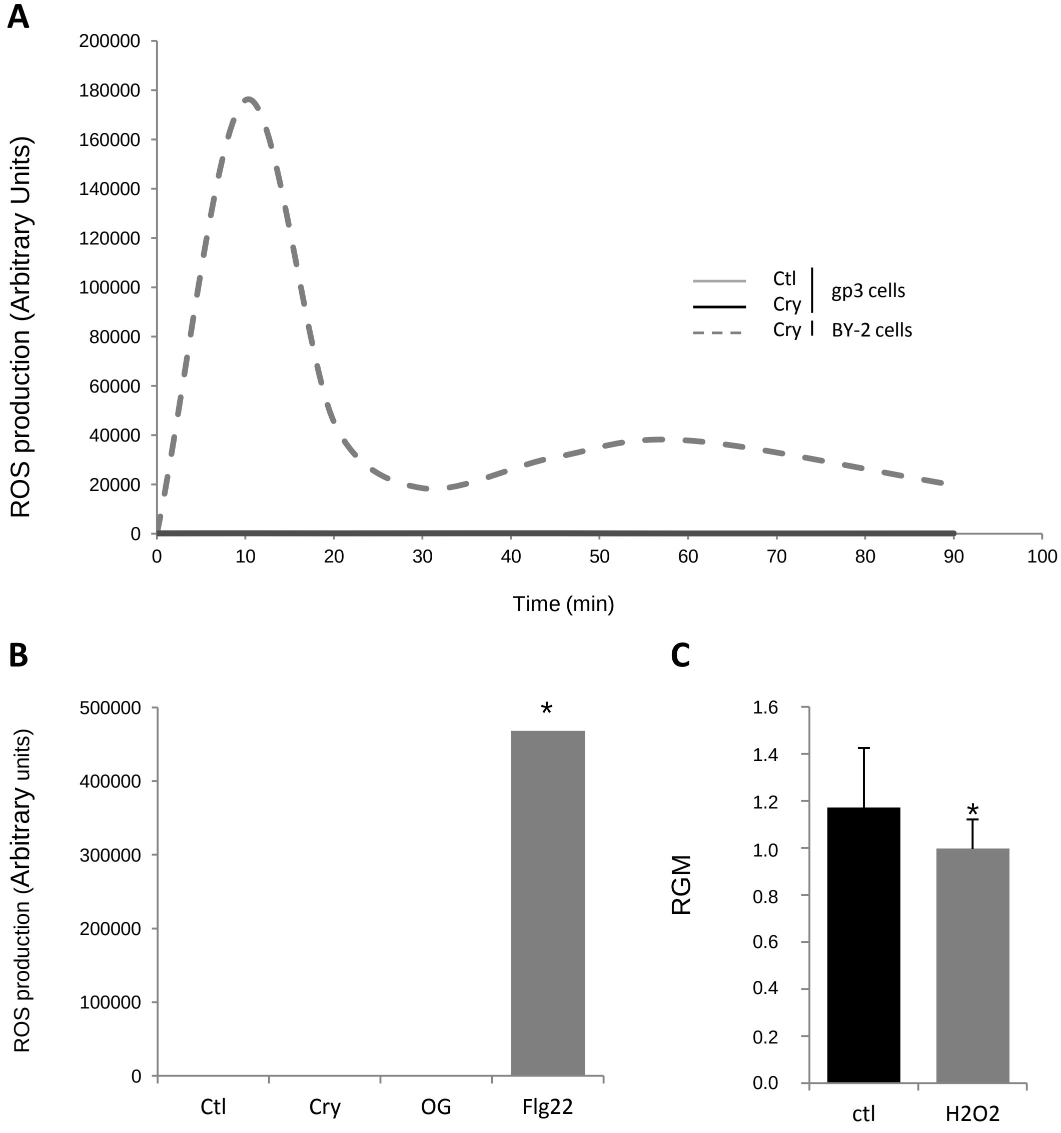

Fig. S4. Relationship between ROS production and PM order in gp3 cell lines. (A) Kinetics of ROS production induced by cryptogein in different tobacco cell lines, wild type (BY-2, used as positive control) and transformed with an antisense construct of *NtrbohD* (gp3). Effect of elicitation (Cry, 50 nM) on ROS production was measured by chemiluminescence in BY-2 cells and gp3 cells. One representative curve was reported (n>6 independent experiments). (B) Effect of elicitors (Cry, 50 nM, OG, 50 ng.ml^{-1} or Flg22, 20 nM) on ROS production of gp3 cells. ROS production (corresponding to the ROS produced after the 10 first min of treatment) was measured by chemiluminescence. We reported one representative value from at least 3 independent experiments. Asterisks indicate a significant difference (p value<0.05). (C) Modification of PM order by exogenous hydrogen peroxide in gp3 cells. Effect of H_2O_2 addition (100 µM) on RGM of tobacco gp3 cells PM. Data shown are mean values ± SEM (n>4 independent experiments). Asterisks indicate a significant difference (p value<0.05).

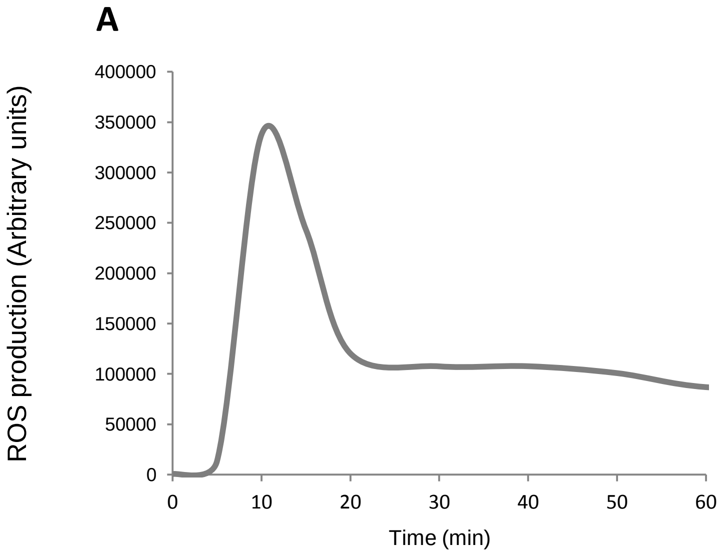

Fig. S5. Kinetics of ROS production induced by cryptogein in tobacco cells. Effect of elicitors (Cry, 50 nM) on ROS production of BY-2 cells was measured by chemiluminescence. (A) One representative curve was reported. (B) ROS production corresponding to the ROS produced as soon as the 5 first min of treatment was reported. Mean values ± SEM (n>6 independent experiments). Asterisks indicate a significant difference (p value<0.05).

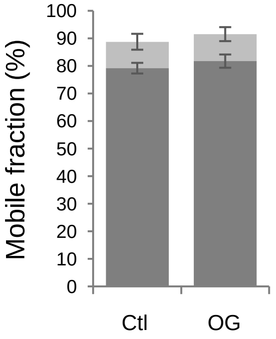

Fig. S6. Effect of oligogalacturonides (OG) on PM fluidity of BY-2 cells. Two FRAP measurements were systematically performed and the mobile fraction was calculated for the two consecutive bleach (dark grey) and re-bleach (light grey) sessions. Mean values ± SEM (n>7 independent experiments).

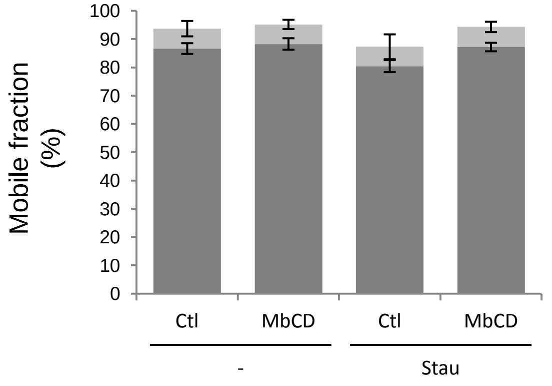

Fig. S7. Influence of sterol depletion on the mobile fraction during two consecutive bleach and re-bleach sessions. Membrane fluidity was measured by FRAP experiments after sterol depletion (15 min of a 5 mM Methylbcyclodextrin treatment, MbCD) and/or phosphorylation inhibition (by a 5 min incubation with 2.5 mM staurosporin, Stau). Two FRAP measurements were systematically performed and the mobile fraction was calculated for the two consecutive bleach (dark grey) and re-bleach (light grey) sessions. The data shown are mean values ± SEM (n>4 independent experiments).

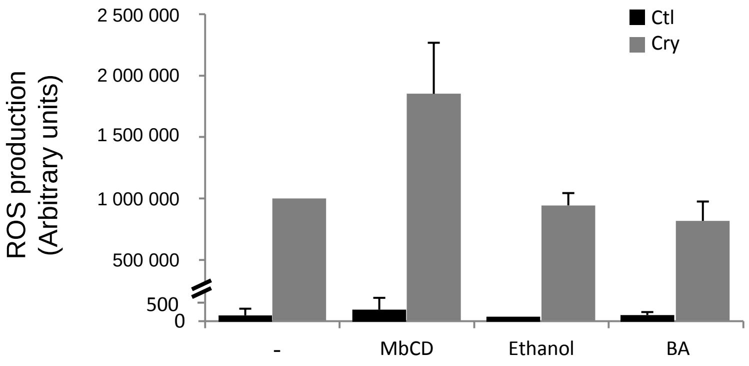

Fig. S8. Fluidizers without sterol trapping capacity are not able to enhance oxidative burst. ROS production (sum of the production during the first 30 min of treatment) was measured after addition of Methylbcyclodextrin (MbCD, 5 mM), ethanol (0.1%) or benzyl alcohol (BA, 20 mM), 15 min prior to cryptogein addition (Cry, 50 nM, grey histogram). Values are compared to control condition without fluidifiant treatment (Ctl, black histogram). Data shown are mean values ± SEM (n>3 experiments).
